# Enhancement of Doxorubicin Efficacy by Bacopaside II in Triple-Negative Breast Cancer Cells

**DOI:** 10.3390/biom15010055

**Published:** 2025-01-03

**Authors:** Sima Kianpour Rad, Kenny K. L. Yeo, Runhao Li, Fangmeinuo Wu, Saifei Liu, Saeed Nourmohammadi, William M. Murphy, Yoko Tomita, Timothy J. Price, Wendy V. Ingman, Amanda R. Townsend, Eric Smith

**Affiliations:** 1 Solid Tumour Group, Basil Hetzel Institute for Translational Health Research, The Queen Elizabeth Hospital, Central Adelaide Local Health Network, Woodville South, Adelaide, SA 5011, Australia; sima.kianpourrad@adelaide.edu.au (S.K.R.); kenny.yeo@adelaide.edu.au (K.K.L.Y.); runhao.li@adelaide.edu.au (R.L.); fangmeinuo.wu@adelaide.edu.au (F.W.); saifei.liu@sa.gov.au (S.L.); saeed.nourmohammadi@adelaide.edu.au (S.N.); yoko.tomita@sa.gov.au (Y.T.); timothy.price@sa.gov.au (T.J.P.); amanda.townsend@sa.gov.au (A.R.T.); 2 Adelaide Medical School, The University of Adelaide, Adelaide, SA 5005, Australia; wendy.ingman@adelaide.edu.au; 3 Department of Surgery-Otolaryngology Head and Neck Surgery, The University of Adelaide and the Basil Hetzel Institute for Translational Health Research, Central Adelaide Local Health Network, Adelaide, SA 5000, Australia; william.murphy@adelaide.edu.au; 4 Medical Oncology, The Queen Elizabeth Hospital, Central Adelaide Local Health Network, Woodville South, Adelaide, SA 5011, Australia; 5 Robinson Research Institute, The University of Adelaide, Adelaide, SA 5005, Australia; 6 Discipline of Surgery, Basil Hetzel Institute for Translational Health Research, The Queen Elizabeth Hospital, Central Adelaide Local Health Network, Woodville South, Adelaide, SA 5011, Australia

**Keywords:** triple-negative breast cancer, molecular subtypes, bacopaside II, *Bacopa monnieri*, doxorubicin, chemotherapy, ATP-binding cassette, P-glycoprotein (*ABCB1*), multidrug resistance protein 1 (*ABCC1*), multidrug resistance protein 3 (*ABCC3*), breast cancer resistance protein (*ABCG2*)

## Abstract

Background: Triple-negative breast cancer (TNBC) is an aggressive subtype with limited treatment options and high resistance to chemotherapy. Doxorubicin is commonly used, but its efficacy is limited by variable sensitivity and resistance. Bacopaside II, a saponin compound, has shown anti-cancer potential. This study evaluates the effects of doxorubicin and bacopaside II, both individually and in combination, across TNBC subtypes to explore mechanisms of resistance and enhanced drug efficacy. Methods: The growth-inhibitory effects of doxorubicin and bacopaside II were assessed in four TNBC cell lines. IC50 values were determined using dose–response assays, and doxorubicin accumulation was measured via spectral flow cytometry. ATP-binding cassette (ABC) transporter expression (*ABCB1*, *ABCC1*, *ABCC3*, and *ABCG2*) was analyzed for correlations with drug sensitivity. In silico docking assessed the binding affinity of bacopaside II to ABC transporters. A 3D culture model simulated drug-resistant TNBC, and combination effects were evaluated with live-cell imaging. Results: Doxorubicin sensitivity varied across TNBC molecular subtypes, correlating to intracellular accumulation. Bacopaside II inhibited growth across subtypes, inducing apoptosis in sensitive cells and necrosis in resistant cells. Bacopaside II increased doxorubicin accumulation, independent of P-glycoprotein (*ABCB1*), possibly through interactions with other ABC transporters. In drug-resistant 3D cultures, bacopaside II maintained efficacy and enhanced doxorubicin accumulation, counteracting ABC transporter-mediated resistance. The doxorubicin and bacopaside II combination showed synergistic growth inhibition. Conclusions: Bacopaside II enhances doxorubicin efficacy in TNBC by increasing drug accumulation and overcoming ABC transporter-mediated resistance, suggesting its potential as an adjuvant in TNBC treatment. These findings support further investigation of bacopaside II, particularly for resistant TNBC subtypes.

## 1. Introduction

Triple-negative breast cancer (TNBC) is a distinct subtype of breast cancer, representing 10–20% of cases and showing a higher prevalence among younger individuals, women of African or Hispanic descent, and those with BRCA1 mutations [1,2,3]. Characterized by the absence of an estrogen receptor, a progesterone receptor, and human epidermal growth factor receptor 2, TNBC is unresponsive to targeted hormonal and HER2 therapies. Molecular classification has further identified distinct TNBC subtypes—basal-like 1, basal-like 2, immunomodulatory, mesenchymal, mesenchymal stem-like, and luminal androgen receptor—each with unique biological and clinical characteristics that could inform personalized treatment strategies [4,5]. Clinically, TNBC is characterized by high invasiveness, metastatic potential, a tendency for relapse, and a generally poor prognosis. Current treatments rely on cytotoxic chemotherapeutics, such as doxorubicin, paclitaxel, and 5-fluorouracil, but their efficacy is often compromised by resistance and toxicity, leading to suboptimal patient outcomes [6,7].

Chemoresistance in TNBC is associated with several mechanisms, including the overexpression of ATP-binding cassette (ABC) transporters [8]. These highly conserved transporters facilitate the translocation of a wide range of substrates across cellular membranes, including lipids, metabolic by-products, xenobiotics, and drugs. The human ABC transporter superfamily includes 48 members, categorized into seven subfamilies (ABCA to ABCG) based on amino acid sequence similarity. Structurally, these transporters are characterized by two transmembrane domains (TMDs), which form the substrate-binding site, and two nucleotide-binding domains (NBDs), which hydrolyze ATP to drive substrate transport. Numerous ABC transporters are implicated in multidrug resistance, enabling cancer cells to expel diverse drugs [9]. Among these, P-glycoprotein (*ABCB1*), multidrug resistance protein 1 (*ABCC1*), multidrug resistance protein 3 (*ABCC3*), breast cancer resistance protein (*ABCG2*), and multidrug resistance protein 8 (*ABCC11*) have been specifically linked to chemoresistance in TNBC [10,11,12,13]. Consequently, targeting ABC transporters represents a promising therapeutic strategy to overcome multidrug resistance in cancer [14,15].

*Bacopa monnieri* extracts have a long history of medicinal use, particularly for memory enhancement and, more recently, as a sedative, with limited adverse effects reported [16,17]. The primary active compounds include saponins such as bacopaside I, bacopaside II, bacopasaponin C, bacoside A, and bacoside A3. Previous studies have shown that *Bacopa monnieri* extracts and their constituents can inhibit P-glycoprotein-mediated drug efflux, with bacopaside II identified as particularly potent [18]. In animal studies, oral administration of the extract reduced intestinal P-glycoprotein expression and transport, while sparing hepatic P-glycoprotein expression [19]. However, it remains unclear whether bacopaside II can inhibit other ABC transporters.

Our prior research has demonstrated that bacopaside II inhibits proliferation, migration, and invasion in breast and colorectal cancer cells and exhibits anti-angiogenic effects in human and mouse endothelial cells [20,21,22,23]. Bacopaside II exerts its antiproliferative effects through cell cycle arrest and apoptosis induction [22], suggesting it may have potential as a novel chemotherapeutic agent. However, it is still unknown whether bacopaside II can enhance the efficacy of other chemotherapeutic agents.

In this study, we aimed to assess whether bacopaside II could potentiate the efficacy of doxorubicin, a commonly used TNBC chemotherapeutic. Specifically, we investigated the impact of bacopaside II on intracellular doxorubicin accumulation in TNBC cells and explored its interactions with ABC transporters known to mediate drug efflux. Given the critical role of ABC transporters in chemoresistance, our findings may offer insights into a novel therapeutic strategy for overcoming drug resistance in TNBC, potentially improving treatment outcomes for this aggressive breast cancer subtype.

## 2. Materials and Methods

### 2.1. Reagents

Bacopaside II analytical standard (≥95% purity by HPLC; CAS no: 382146-66-9, CAT#44698), paclitaxel (≥95% purity by HPLC; CAS no: 33069-62-4, CAT#T7402), 5-fluorouracil (≥99% purity by HPLC; CAS no: 51-21-8, CAT#F6627), methanol (≥99% purity by HPLC; CAS no: 67-56-1), and dimethyl sulfoxide Hybri-Max (DMSO; ≥99.7% purity; CAS no: 67-68-5, CAT#D2650) were purchased from Sigma-Aldrich (St. Louis, MO, USA). Doxorubicin hydrochloride was sourced from Pfizer Australia Pty, Ltd. (Sydney, NSW, Australia).

### 2.2. TNBC Cell Lines

The TNBC cell lines DU4475, HCC1143, MDA-MB-231, and MDA-MB-453 were obtained from the American Type Culture Collection (ATCC, Manassas, VA, USA). MDA-MB-231 and MDA-MB-453 cells were cultured in Dulbecco’s Modified Eagle’s Medium (DMEM; Thermo Fisher Scientific, Waltham, MA, USA), while DU4475 and HCC1143 cells were cultured in RPMI-1640 (Thermo Fisher Scientific). All media were supplemented with 10% heat-inactivated fetal bovine serum (FBS; Corning, Corning, NY, USA), 200 U/mL penicillin, and 200 μg/mL streptomycin (Thermo Fisher Scientific). Cells were maintained at 37 °C in a humidified atmosphere containing 5% CO_2_. Mycoplasma contamination was ruled out using the MycoAlert Mycoplasma Detection Kit (Lonza, Basel, Switzerland).

### 2.3. Scaffold-Free 3D Suspension Spheroid Culture

MDA-MB-231 cells were cultured as scaffold-free 3D suspension spheroids by seeding 4 × 10⁴ cells per well into ultra-low attachment 6-well plates (Corning Incorporated, Tewksbury, MA, USA, CAT#3471). Cells were maintained in MammoCult Basal Medium supplemented with 10% MammoCult Proliferation Supplement, 4 μg/mL heparin, and 0.48 μg/mL hydrocortisone (STEMCELL Technologies, Vancouver, BC, Canada, CAT#05620), along with 200 U/mL penicillin and 200 μg/mL streptomycin. The 3D cultures were passaged weekly for up to four weeks. For passaging, cell aggregates were dissociated into single cells using 2% (*w*/*v*) EDTA in DPBS (Thermo Fisher Scientific), washed in MammoCult medium, and reseeded at the initial density of 4 × 10⁴ cells per well. The cell aggregates were dissociated into single cells for experiments.

### 2.4. Drug Sensitivity Assays

Cells were seeded at a density of 3.3 × 10³ cells per well in 96-well flat-bottom plates (Corning, CAT#3599). For DU4475 cells, plates were pre-coated with 0.01% (*w*/*v*) poly-L-orthithine (Sigma-Aldrich, CAT#P4957) to promote cell attachment. Following overnight incubation, cells were treated with varying concentrations of the test drugs for five days. The drugs were prepared in a final vehicle concentration (*v*/*v*) of 2% methanol for bacopaside II, 0.01% DMSO for paclitaxel, and 0.13% DMSO for 5-fluorouracil. After the treatment period, cell proliferation was assessed using a crystal violet staining assay, as previously described [22]. Additionally, cell proliferation was continuously monitored by measuring the percentage of the well area occupied by cells (percent confluence) using the label-free Incucyte Proliferation Assay on an Incucyte S3 Live-Cell Analysis System (Sartorius AG, Göttingen, Germany).

### 2.5. Evaluation of Cell Membrane Integrity Using Propidium Iodide and Apoptosis Using Caspase-3/7 Assays

Cells were seeded at a density of 3.3 × 10³ cells per well in 96-well plates, incubated overnight, and treated with increasing concentrations of bacopaside II in a 2% methanol vehicle. Media were supplemented with either 2.5 µg/mL propidium iodide (PI) (Sigma-Aldrich) to assess membrane integrity or 0.5 µM CellEvent Caspase-3/7 Green Detection Reagent (Thermo Fisher Scientific) to detect apoptosis. Drozitumab was freshly prepared by mixing equal volumes of 100 ng/µL drozitumab (Thermo Fisher Scientific, CAT#MA5-41916, RRID:AB_2911059) and 100 ng/µL affinity-purified goat anti-human IgG Fcγ fragment (Jackson Immunoresearch Laboratories Inc., West Grove, PA, USA, CAT#109-005-008, RRID:AB_2337534), incubating for 30 min at 4 °C, and diluting to a final concentration of 100 ng/mL in culture medium [24]. Staurosporine (Sigma-Aldrich, CAT#S5921) was used at a final concentration of 2.5 µM [25]. Live-cell imaging was conducted at 2-h intervals over 48 h using an Incucyte S3 Live-Cell Analysis System. Phase-contrast and red or green fluorescence images were captured to monitor confluency and PI-positive or caspase-3/7-positive cells. Data were presented as the average number of PI-positive or caspase-3/7-positive cells per image, along with their 95% confidence intervals, across the time course. Comparisons were made to the respective vehicle controls for each experiment.

### 2.6. Doxorubicin Accumulation Assay

Cells were seeded at a density of 2.1 × 10^5^ per well in 12-well plates and incubated overnight. After incubation, cells were treated with increasing concentrations of doxorubicin for 24 h. Following treatment, cells were washed three times with Dulbecco’s Phosphate Buffered Saline (DPBS; Thermo Fisher Scientific), dissociated using 2% (*w*/*v*) ethylenediaminetetraacetic acid EDTA (Sigma-Aldrich) at 37 °C, and resuspended in DMEM supplemented with 10% FBS. Viability was assessed using BD Horizon Fixable Viability Stain 780 (FVS780) (BD Biosciences, Ashland, OR, USA). Intracellular doxorubicin levels in viable cells were quantified using a Cytek Aurora spectral flow cytometer (Cytek Bioscience, Fremont, CA, USA) [26]. Data were acquired from a minimum of 50,000 single-cell events per sample, and intracellular doxorubicin content was calculated as the geometric mean fluorescence intensity (GMFI) using FlowJo software v10.10.0 (BD Biosciences).

### 2.7. RNA Extraction, cDNA Synthesis, and Analysis of Gene Expression by Quantitative TaqMan PCR

Cells were seeded at 5 × 10^5^ cells per well in 6-well plates (Corning) and incubated overnight. RNA was isolated using the PureLink RNA Mini Kit (Thermo Fisher Scientific). Total RNA was quantified using a NanoDrop 2000 spectrophotometer (Thermo Fisher Scientific), and 200 ng was reverse-transcribed using the SuperScript IV VILO Master Mix (Thermo Fisher Scientific) in a final volume of 20 μL. Transcript expression was quantified using duplex TaqMan Gene Expression Assays (Thermo Fisher Scientific). Each assay included a reference gene, human hypoxanthine phosphoribosyltransferase-1 (*HPRT1*; Hs99999909_m1), and a gene of interest: Nanog homeobox pseudogene-1 (*NANOG*; Hs04399610_g1), Octamer-binding transcription factor-4 (*OCT4*; Hs00742896_s1), ATP binding cassette subfamily B member 1 (*ABCB1*; Hs00184500_m1), ATP binding cassette subfamily C member 1 (*ABCC1;* Hs01561483_m1), ATP binding cassette subfamily C member 3 (*ABCC3*; Hs00358656_m1), or ATP binding cassette subfamily G member 2 (*ABCG2*; Hs01053790_m1). Each 10 μL reaction consisted of 1 μL of cDNA, 0.5 μL of the primer for the gene of interest, 0.5 μL of the reference gene primer, 5 μL TaqMan Fast Advanced Master Mix, and 3 μL of PCR-grade water. Amplification was performed using a ViiA 7 Real-Time PCR System (Thermo Fisher Scientific) with an initial activation step of 2 min at 95 °C, followed by 40 cycles of 1 s at 95 °C and 20 s at 60 °C. Gene expression was normalized to the *HPRT1* reference gene using the 2^−ΔCt^ method. For comparisons relative to the 2D condition (relative quantification, RQ), gene expression was calculated using the 2^−ΔΔCt^ method. All data are presented as the mean ± standard deviation (SD) of three technical replicates from three independent experiments.

### 2.8. Western Immunoblot

MDA-MB-231 cells were seeded at 5 × 10^5^ cells per well in 6-well plates (Corning) and incubated overnight. Cells were then treated with 0 µM (2% methanol vehicle control), 15 µM, 20 µM, and 30 µM bacopaside II for 4 h. Following treatment, cells were lysed using RIPA Lysis and Extraction Buffer supplemented with the Halt Protease Inhibitor Cocktail and the Halt Phosphatase Inhibitor Cocktail (Thermo Fisher Scientific), homogenized by passing through a 26-gauge needle, and centrifuged at 17,000× *g* for 15 min at 4 °C to remove insoluble cell debris. Total protein concentrations were determined using a Bio-Rad Protein Assay (Bio-Rad, Hercules, CA, USA), and 50 µg was resolved by denaturing electrophoresis using 4–15% Mini-PROTEAN TGX Stain-Free precast gels and transferred to 0.2 μm polyvinylidene difluoride membranes using a Trans-Blot Turbo Transfer System (Bio-Rad). Membranes were blocked with either 5% skim milk (for anti-MLKL) or 5% BSA (for anti-pS358-MLKL) in Tris-buffered saline supplemented with 0.05% (*v*/*v*) Tween-20 (TBST) for at least 1 h. Membranes were incubated at 4 °C overnight with 1:1000 dilution anti-MLKL (clone [EPR17514], ab184718) in TBST with 0.1% milk, or 1:1000 anti-pS358-MLKL ([EPR9514], ab187091) in TBST with 2% BSA. Membranes were washed 5 times with TBST, incubated with a 1:2000 goat anti-rabbit (H+L)–HRP conjugated secondary antibody (Bio-Rad) for 1 h, and visualized using the Clarity Western ECL Blotting Substrate with a ChemiDoc Gel Imaging System (Bio-Rad). The MLKL and phospho-MLKL (pMLKL) bands were quantified and normalized to total protein levels using Image Lab Software v6.0.1 (Bio-Rad).

### 2.9. Molecular Docking Analysis

The SMILES structures of bacopaside II, cyclosporin A, and elacridar were obtained from PubChem (https://pubchem.ncbi.nlm.nih.gov). Protein structures for the ABC transporters ABCB1 (P08183), ABCC1 (P33527), ABCC3 (O15438), and ABCG2 (A1LUE4) were retrieved from the AlphaFold Protein Structure Database (https://alphafold.ebi.ac.uk) [27,28]. Molecular docking was conducted using Autodock Vina (https://vina.scripps.edu; version vina_1.2.3_mac_x86_64) to predict the Gibbs free energy of protein–ligand binding (kJ/mol) through flexible ligand docking simulations [29,30]. The docking was performed within defined grids at specified interaction sites on each protein, using the following parameters: --size_x 30 --size_y 30 --size_z 30 --cpu 6 --exhaustiveness 36 --seed 100. UCSF ChimeraX (https://www.cgl.ucsf.edu/chimerax/; version 1.6.dev202301202101 (accessed on 20 January 2023)) was used to visualize the 3D structures of the ABC transporter proteins and the ligands [31,32].

### 2.10. Statistical Analysis

All statistical analyses were performed using Prism 10 for macOS (Version 10.4.0 (527), 23 October 2024; GraphPad Software Inc., La Jolla, CA, USA).

## 3. Results

### 3.1. Doxorubicin-Induced Growth Inhibition in TNBC Cells

**Figure 1:**
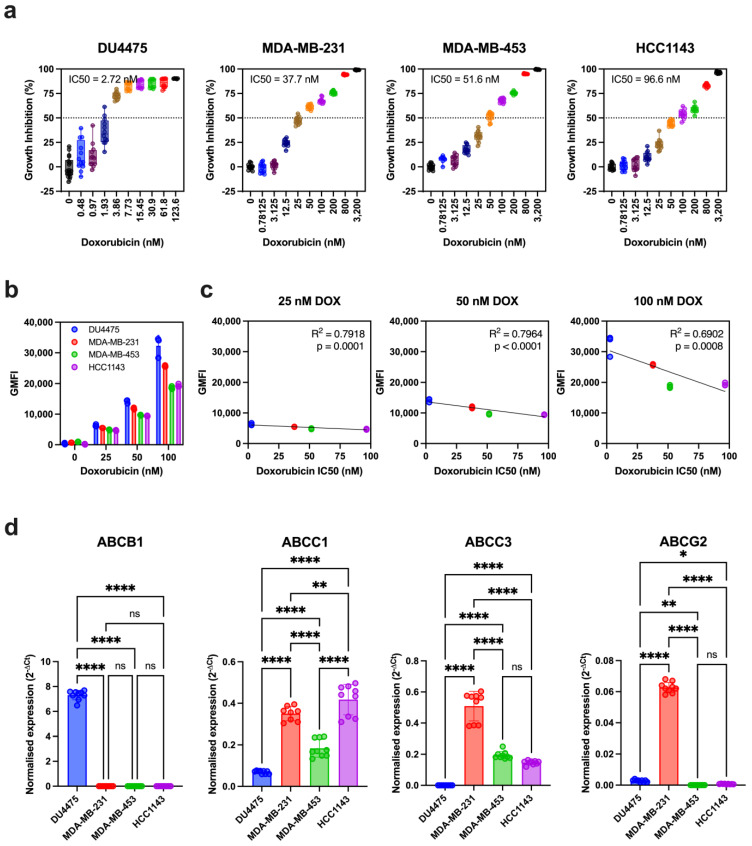
Doxorubicin sensitivity, accumulation, and ABC transporter gene expression in TNBC cell lines. (**a**) TNBC cell lines (DU4475, MDA-MB-231, MDA-MB-453, and HCC1143) were treated with doxorubicin for five days, followed by crystal violet staining to assess growth inhibition. Data are presented as individual values with median, interquartile range (25th–75th percentiles), and whisker plots from at least six replicates, normalized to the vehicle control. (**b**) Doxorubicin accumulation was evaluated in TNBC cell lines after 24 h of treatment with increasing doxorubicin concentrations (0, 25, 50, and 100 nM). Intracellular doxorubicin levels were quantified via flow cytometry, with geometric mean fluorescence intensity (GMFI) representing doxorubicin accumulation. Data are shown as individual values with standard deviations (SD) from three independent experiments. (**c**) Correlation between doxorubicin IC50 values and intracellular GMFI across TNBC cell lines: DU4475 (blue), MDA-MB-231 (red), MDA-MB-453 (green), and HCC1143 (purple). Data points represent GMFI values from three separate experiments using 25 nM, 50 nM, and 100 nM doxorubicin (DOX). (**d**) Transcript expression levels of ABC transporters (*ABCB1*, *ABCC1*, *ABCC3*, and *ABCG2*) were measured in untreated TNBC cell lines using TaqMan Assays. Expression levels were normalized to the *HPRT1* reference gene using the 2^−ΔCt^ method. Data represent the mean ± SD of three technical replicates across three independent cultures and were analyzed using ordinary one-way ANOVA with Holm–Sidak’s multiple comparisons test. Statistical significance is indicated as **** *p* < 0.0001, ** *p* < 0.01, * *p* < 0.05, not significant (ns; *p* > 0.05).

The growth-inhibitory effects of doxorubicin were assessed across a panel of cell lines representing four common TNBC molecular subtypes: immunomodulatory (DU4475), mesenchymal stem-like (MDA-MB-231), luminal androgen receptor (MDA-MB-453), and basal-like 1 (HCC1143) [4]. The half-maximal inhibitory concentration (IC50) of doxorubicin varied widely among these cell lines, highlighting differential sensitivity to the drug. The IC50 values were 2.72 nM (95% CI: 2.42–3.07 nM) for DU4475, 37.7 nM (95% CI: 35.2–40.3 nM) for MDA-MB-231, 51.6 nM (95% CI: 49.1–54.3 nM) for MDA-MB-453, and 96.6 nM (95% CI: 89.1–105 nM) for HCC1143 (Figure 1a).

Kinetic live-cell imaging of MDA-MB-231 cells further revealed that doxorubicin’s growth-inhibitory effects were not evident within the first 24 h post-treatment, even at concentrations as high as 3.2 µM (Figure S1). These findings suggest that doxorubicin’s efficacy in TNBC cells may depend on prolonged exposure.

To evaluate whether doxorubicin sensitivity correlated with intracellular accumulation, we measured doxorubicin’s intrinsic fluorescence in viable cells using spectral flow cytometry (Figure 1b and Figure S2). As expected, doxorubicin accumulation increased in a concentration-dependent manner, with higher intracellular levels observed at greater drug concentrations. DU4475, the most sensitive cell line, exhibited the highest intracellular doxorubicin levels, whereas MDA-MB-453 and HCC1143, the least sensitive cell lines, showed the lowest accumulation. A significant correlation was observed between doxorubicin accumulation and IC50 values across the cell lines, with linear regression R-squared values of 0.7918 (*p* = 0.0001), 0.7964 (*p* < 0.0001), and 0.6902 (*p* = 0.0008) at concentrations of 25, 50, and 100 nM, respectively (Figure 1c).

We investigated the relationship between doxorubicin accumulation, sensitivity, and the transcript expression of *ABCB1*, which encodes P-glycoprotein, a key efflux protein involved in doxorubicin transport (Figure 1d). Interestingly, *ABCB1* expression was absent or barely detectable in MDA-MB-231, MDA-MB-453, and HCC1143, the cell lines with the lowest sensitivity and intracellular accumulation of doxorubicin. Conversely, *ABCB1* expression was significantly higher in DU4475, the cell line with the highest sensitivity and intracellular doxorubicin levels. These findings suggest that *ABCB1* expression alone is insufficient to fully explain the differences in doxorubicin accumulation and sensitivity across these TNBC cell lines.

To investigate the potential involvement of other ABC transporters associated with breast cancer chemoresistance, we analysed the expression of *ABCC1*, *ABCC3*, and *ABCG2* (Figure 1d). Compared to the high normalized expression of *ABCB1* in DU4475, the expression levels of *ABCC1* and *ABCC3* were moderate, while *ABCG2* expression was markedly lower or undetectable across the cell lines. *ABCC1* expression was highest in HCC1143, followed by MDA-MB-231 and then MDA-MB-453, and was the lowest in DU4475. *ABCC3* expression was most prominent in MDA-MB-231, followed by MDA-MB-453 and HCC1143, with no detectable expression in DU4475. For *ABCG2*, expression was highest in MDA-MB-231 but low or undetectable in DU4475, HCC1143, and MDA-MB-453.

Notably, no correlation was observed between the individual expression levels of *ABCB1*, *ABCC1*, *ABCC3*, or *ABCG2* and doxorubicin accumulation or sensitivity. This highlights the complexity of intracellular drug accumulation mechanisms and suggests potential functional redundancy among members of the ABC transporter family.

### 3.2. Bacopaside II-Induced Growth Inhibition of TNBC Cell Lines Is Mediated by Apoptosis and Necrosis

**Figure 2:**
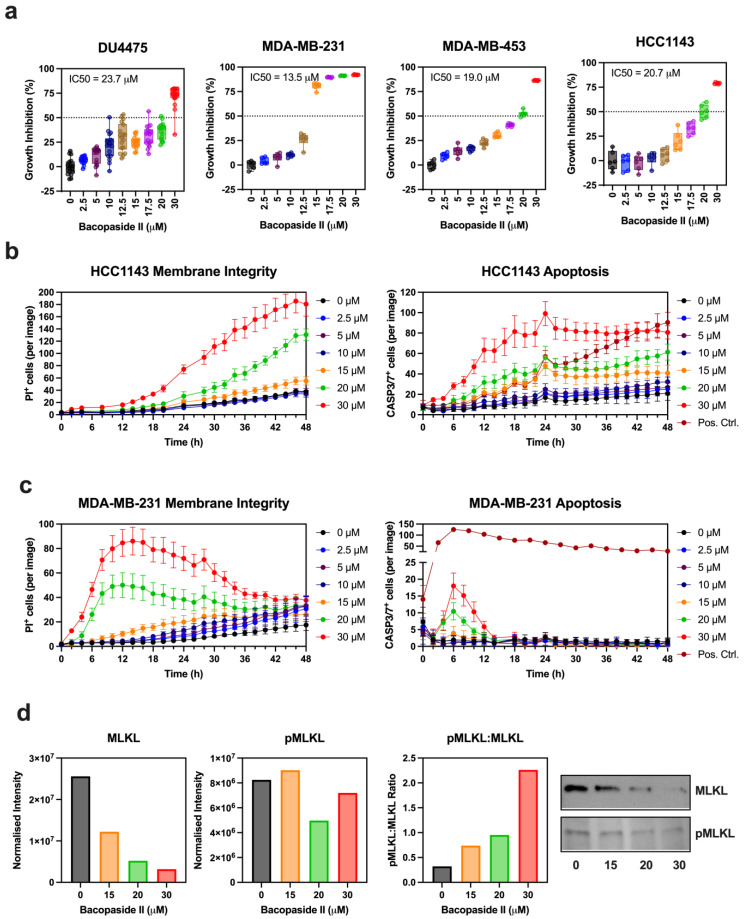
Bacopaside II sensitivity and induction of cell death in TNBC cell lines. (**a**) TNBC cell lines (DU4475, MDA-MB-231, MDA-MB-453, and HCC1143) were treated with bacopaside II for five days, followed by crystal violet staining to assess growth inhibition. Data are presented as individual values with median, interquartile range (25th–75th percentiles), and whisker plots from at least six replicates, normalized to the vehicle control (2% methanol). (**b**) Kinetic live-cell imaging of HCC1143 cells showing the number of propidium iodide (PI)-positive cells (loss of membrane integrity; left panel) and caspase-3/7-positive cells (apoptosis; right panel) measured every 2 h over 48 h. Staurosporine (2.5 µM) was used as a positive control for apoptosis. Data represent the mean ± 95% confidence interval (CI) of up to six replicates. (**c**) Kinetic live-cell imaging of MDA-MB-231 cells showing the number of PI-positive cells (loss of membrane integrity; left panel) and caspase-3/7-positive cells (apoptosis; right panel) measured every 2 h over 48 h. Drozitumab (100 ng/mL) served as a positive control for apoptosis. Data represent the mean ± 95% CI of up to six replicates. (**d**) Western blot analysis of MLKL and phosphorylated MLKL (pMLKL) following 4 h of treatment with 0, 15, 20, and 30 µM bacopaside II. Band intensities were normalized to total protein levels.

We assessed bacopaside II’s growth-inhibitory effects across the TNBC cell lines (Figure 2). The IC50 values were as follows: 23.7 µM for DU4475 (95% CI: 21.1–28.6 µM), 13.5 µM for MDA-MB-231 (95% CI: 13.3–13.8 µM), 19.0 µM for MDA-MB-453 (95% CI: 18.3–19.7 µM), and 20.7 µM for HCC1143 (95% CI: 19.9–21.6 µM) (Figure 2a). The minimum inhibitory concentrations were 10 µM for DU4475 (*p* = 0.0018), 15 µM for MDA-MB-231 (*p* = 0.0174), 15 µM for MDA-MB-453 (*p* = 0.0082), and 20 µM for HCC1143 (*p* = 0.0059).

We then evaluated bacopaside II’s ability to induce apoptosis in TNBC cell lines with differing resistance to apoptotic stimuli: HCC1143 (moderate resistance) and MDA-MB-231 (high resistance). Kinetic live-cell imaging was performed with fluorescence markers, using caspase-3/7 activation as an apoptosis indicator and propidium iodide (PI) for membrane integrity (Figure 2b). In HCC1143 cells, bacopaside II treatment led to a rapid increase in caspase-3/7-positive cells, which preceded the loss of membrane integrity. Treatment with 30, 20, and 15 µM bacopaside II caused significant increases in caspase-3/7-positive cells at 2, 6, and 8 h, respectively, compared to the vehicle control. Loss of membrane integrity followed at 4, 18, and 28 h for 30, 20, and 15 µM treatments, respectively. These findings suggest that in HCC1143 cells, bacopaside II induces apoptosis followed by membrane integrity loss, consistent with late-stage apoptosis.

In MDA-MB-231 cells, bacopaside II treatment induced a rapid, concentration-dependent loss of membrane integrity that preceded apoptosis (Figure 2c). Treatment with 30, 20, and 15 µM bacopaside II significantly increased PI-positive cells at 2, 4, and 8 h, respectively, compared to the vehicle control. However, only the 30 and 20 µM doses significantly increased caspase-3/7-positive cells, observed first at 4 h and peaking at 6 h. At 6 h, the percentage of PI-positive cells exceeded caspase-3/7-positive cells, suggesting that loss of membrane integrity dominated over apoptosis at higher doses (Figure S3). These results indicate that in MDA-MB-231 cells, bacopaside II growth inhibition is primarily driven by loss of membrane integrity, consistent with necrotic cell death.

To further investigate bacopaside II-induced necrotic cell death, we performed Western blot analysis for the necroptosis marker mixed lineage kinase domain-like (MLKL) and its activated form, phospho-MLKL (pMLKL). Bacopaside II induced a dose-dependent reduction in MLKL without substantially altering pMLKL levels, though it increased the pMLKL-to-MLKL ratio (Figure 2d and Figure S4). Together, these findings suggest that bacopaside II may promote necroptosis in the apoptosis-resistant TNBC cell line MDA-MB-231. 

### 3.3. Bacopaside II Increases Intracellular Accumulation of Doxorubicin

**Figure 3:**
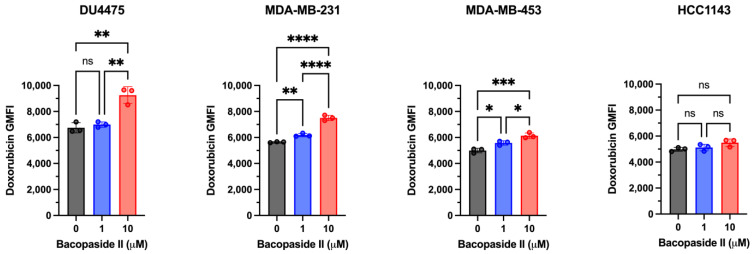
Effect of bacopaside II on doxorubicin accumulation. TNBC cell lines were co-treated with 25 nM doxorubicin and 0 µM, 1 µM, or 10 µM bacopaside II for 24 h. Intracellular doxorubicin levels in viable cells were quantified using flow cytometry. Data represent the mean ± SD of doxorubicin geometric mean fluorescence intensity (GMFI) from three individual experiments. Statistical significance was calculated using ordinary one-way ANOVA followed by Holm–Sidak’s multiple comparisons test. * *p* < 0.05, ** *p* < 0.01, *** *p* < 0.001, **** *p* < 0.0001, ns—*p* > 0.05.

To investigate whether bacopaside II enhances the intracellular accumulation of doxorubicin in TNBC cell lines, cells were treated with non-cytotoxic concentrations of bacopaside II (1 and 10 µM) and compared to vehicle controls (Figure 3 and Figure S5).

When co-treated with 25 nM doxorubicin and 1 µM bacopaside II, significant increases in intracellular doxorubicin levels were observed in MDA-MB-231 and MDA-MB-453, but not in DU4475 or HCC1143. At a higher concentration of bacopaside II (10 µM), significant increases in doxorubicin accumulation were detected in DU4475, MDA-MB-231, and MDA-MB-453. However, HCC1143 cells showed no significant changes in doxorubicin accumulation under any of these treatment conditions.

Interestingly, in DU4475 cells, increasing the concentration of doxorubicin from 25 nM to 50 nM or 100 nM eliminated the bacopaside II-induced enhancement of doxorubicin accumulation (Figure S5). This result suggests that higher concentrations of doxorubicin alone may inhibit doxorubicin efflux in this particularly sensitive cell line, potentially masking the effect of bacopaside II.

To investigate the mechanism by which bacopaside II enhances doxorubicin accumulation in TNBC cells, we conducted in silico docking analyses to evaluate its binding affinity to the substrate-binding transmembrane domain (TMD) and ATPase nucleotide-binding domain (NBD) of ABC transporter proteins (Table 1). Bacopaside II exhibited a strong binding affinity to the TMD of ABCB1 (−8.361 kcal/mol), exceeding the affinities of both first- and third-generation inhibitors, cyclosporin A (−7.324 kcal/mol) and elacridar (−8.177 kcal/mol). For ABCC1 (−7.475 kcal/mol) and ABCC3 (−6.673 kcal/mol), bacopaside II showed higher affinities compared to cyclosporin A (−4.805 and -2.972 kcal/mol, respectively) but lower affinities than elacridar (−11.32 and −10.56 kcal/mol). However, its binding affinity to the ABCG2 TMD (−6.454 kcal/mol) was lower than both cyclosporin A (−6.690 kcal/mol) and elacridar (−8.309 kcal/mol).

For the NBD, bacopaside II demonstrated favorable binding to ABCB1 (−7.864 kcal/mol), surpassing cyclosporin A (2.592 kcal/mol) and matching elacridar (−7.730 kcal/mol). Similarly, bacopaside II displayed higher binding affinities to the NBD of ABCC1 (−6.864 kcal/mol), ABCC3 (−6.356 kcal/mol), and ABCG2 (−7.349 kcal/mol) compared to cyclosporin A (−6.779, −6.084, and −5.558 kcal/mol, respectively). However, its affinities for these NBDs remained lower than those of elacridar (−7.514, −7.502, and −7.989 kcal/mol, respectively).

In summary, these results suggest that bacopaside II interacts favorably with both the TMD and NBD of various ABC transporters and may serve as a more effective inhibitor than cyclosporin A. However, its inhibitory efficacy remains below that of the third-generation inhibitor elacridar.

### 3.4. Bacopaside II’s Modulatory Effects in Doxorubicin-Resistant Cells

**Figure 4:**
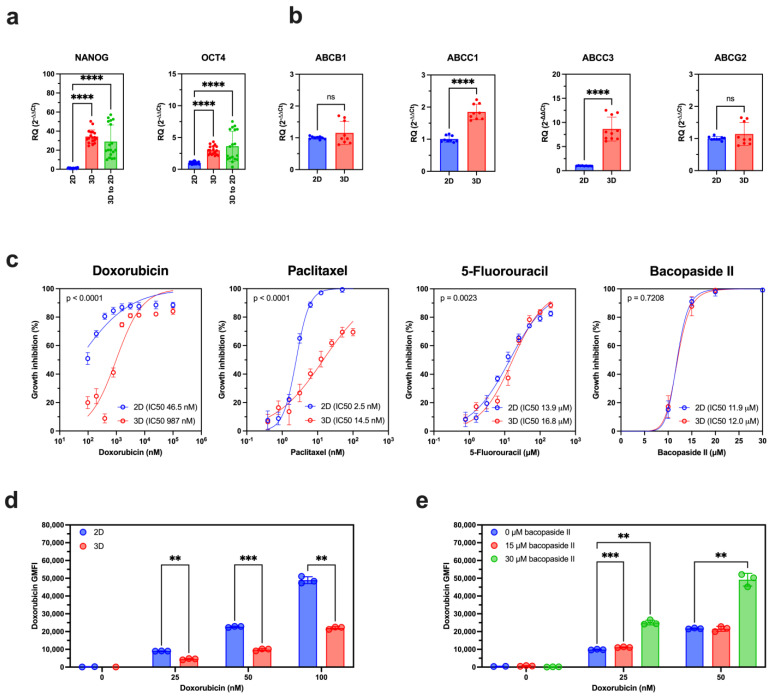
Bacopaside II overcomes multidrug resistance induced by 3D culture of MDA-MB-231 cells. (**a**) Transcript levels of stem cell markers *NANOG* and *OCT4* in MDA-MB-231 cells cultured as an adherent monolayer (2D), in scaffold-free 3D suspension for 4 weeks (3D), or after transitioning from 3D suspension back to adherent monolayer culture for 5 days (3D to 2D). Gene expression was normalized to the reference gene *HPRT1* and calculated relative to the 2D condition using the 2^−ΔΔCt^ method. Data are presented as mean ± SD from three technical replicates across three independent cultures and analyzed using the Kruskal–Wallis test with Dunn’s multiple comparisons test. (**b**) Expression of ABC transporters *ABCB1*, *ABCC1*, *ABCC3*, and *ABCG2* in MDA-MB-231 cells cultured as a 2D monolayer or as a 3D suspension culture for 4 weeks. Data represent mean ± SD from three technical replicates across three independent cultures and were analyzed using the Mann–Whitney test. (**c**) Dose–response curves of 2D-cultured and 4-week 3D-cultured cells following 5 days of treatment with doxorubicin, paclitaxel, 5-fluorouracil, or bacopaside II. Data are presented as mean ± 95% CI from six replicates. (**d**) Intracellular doxorubicin accumulation, measured as geometric mean fluorescence intensity (GMFI) by flow cytometry, in 2D- and 3D-cultured cells after 24 h of treatment with 0, 25, 50, or 100 nM doxorubicin. Data represent mean ± SD from three individual experiments and were analyzed using a mixed-effects model with Geisser–Greenhouse correction and Sidak’s multiple comparisons test. (**e**) Doxorubicin GMFI in 3D-cultured cells following 24-h co-treatment with 0 µM (2% methanol vehicle control), 15 µM, or 30 µM bacopaside II and 0, 25, or 50 nM doxorubicin. Data represent mean ± SD from three individual experiments and were analyzed using a mixed-effects model with Geisser–Greenhouse correction and Sidak’s multiple comparisons test. Statistical significance is indicated as **** *p* < 0.0001, *** *p* < 0.001, ** *p* < 0.01, ns—*p* > 0.05.

To evaluate whether bacopaside II could overcome acquired resistance to conventional chemotherapeutics, MDA-MB-231 cells were cultured as 3D spheroid suspensions, a model known to exhibit upregulated ABCC3 expression and enhanced drug resistance [33]. We observed that this 3D culture model promoted a stem cell-like phenotype, as evidenced by significant increases in the transcript expression of stem cell markers *NANOG* and *OCT4* within just one week of 3D culture (Figure 4a and Figure S4). *NANOG* and *OCT4* expression rose 33.2-fold (*p* < 0.0001) and 2.0-fold (*p* < 0.0001), respectively, by week four (Figure 4a). Importantly, these stem cell-like properties persisted for at least one week after transitioning back to adherent culture (3D to 2D), with *NANOG* and *OCT4* levels remaining elevated compared to adherent 2D-cultured cells (Figure 4a).

We next evaluated the expression of ABC transporters associated with chemotherapeutic resistance. While *ABCB1* or *ABCG2* levels remained unchanged, *ABCC1* and *ABCC3* expression significantly increased following 3D culture, showing 1.8-fold and 8.6-fold elevations, respectively, after four weeks (*p* < 0.0001; Figure 4b). These findings align with previously reported studies [33,34].

To assess chemotherapeutic sensitivity, we compared IC50 values for doxorubicin, paclitaxel, and 5-fluorouracil in 2D- and 3D-cultured cells (Figure 4c). The 3D-cultured cells displayed significant increases in IC50 values, suggesting acquired multi-drug resistance. For doxorubicin, the IC50 increased from 46.5 nM (95% CI 29.4 to 65.4) in 2D cultures to 987 nM (95% CI 807 to 1212) in 3D cultures (*p* < 0.0001). Similarly, the IC50 for paclitaxel rose from 2.5 nM (95% CI 2.4 to 2.6) in 2D to 14.5 nM (95% CI 12.7 to 16.7) in 3D (*p* < 0.0001), and from 13.9 μM (95% CI 12.5 to 15.4) in 2D to 16.8 μM (95% CI 15.1 to 18.7) in 3D (*p* = 0.0023) for 5-fluorouracil. In contrast, bacopaside II sensitivity remained unchanged, with IC50 values of 11.9 µM (95% CI: 11.6–12.2) in 2D cultures and 12.0 µM (95% CI: 11.2–13.0) in 3D cultures (*p* = 0.7208). This stability suggests that bacopaside II may circumvent the acquired resistance mechanisms associated with 3D culture conditions.

We investigated whether 3D-cultured MDA-MB-231 cells exhibit altered drug efflux by measuring intracellular doxorubicin levels. Flow cytometry revealed that doxorubicin accumulation was significantly reduced in 3D-cultured cells compared to 2D-cultured cells after treatment with 25 nM, 50 nM, or 100 nM doxorubicin (*p* < 0.01) (Figure 4d). This reduction indicates enhanced drug efflux capacity in the 3D-cultured cells.

To evaluate whether bacopaside II could counteract this effect, doxorubicin accumulation was measured in 3D-cultured cells co-treated with bacopaside II. Co-treatment with 30 µM bacopaside II significantly increased doxorubicin accumulation at both 25 nM and 50 nM doxorubicin concentrations (*p* < 0.01), whereas 15 µM bacopaside II led to a slight increase when combined with 25 nM doxorubicin (Figure 4e). 

These findings clearly demonstrate that bacopaside II enhances intracellular doxorubicin accumulation in 3D-cultured cells, effectively mitigating the drug efflux associated with the resistant 3D culture model.

### 3.5. Combined Effects of Doxorubicin and Bacopaside II on Cell Proliferation

**Figure 5:**
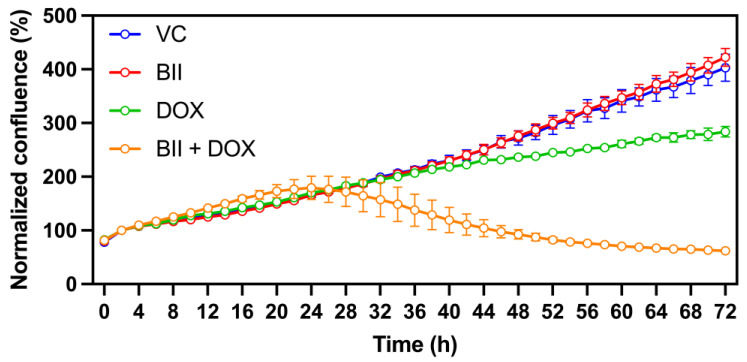
Synergistic inhibition of MDA-MB-231 cell proliferation by combined treatment with doxorubicin and bacopaside II. MDA-MB-231 cells were treated with the vehicle control (VC, 2% methanol), 10 µM bacopaside II (BII), 100 nM doxorubicin (DOX), or a combination of 10 µM bacopaside II and 100 nM doxorubicin (BII + DOX). Cell growth was monitored every 2 h over 72 h using kinetic live-cell imaging, with confluence measurements normalized to the 2-h time point. Data represent the mean ± SD of up to 12 replicates.

Lastly, we assessed the combined effect of bacopaside II and doxorubicin on cell proliferation using kinetic live-cell imaging (Figure 5). Treatment with 10 µM bacopaside II alone had no significant impact on MDA-MB-231 cell growth, while 100 nM doxorubicin caused moderate growth inhibition after 48 h. Strikingly, the combination of bacopaside II and doxorubicin resulted in substantial growth inhibition as early as 32 h. These results highlight a synergistic interaction between bacopaside II and doxorubicin, significantly enhancing growth inhibition in this TNBC cell line.

## 4. Discussion

This study investigates the potential of bacopaside II, a saponin derived from *Bacopa monnieri*, to enhance the efficacy of doxorubicin in treating TNBC, a particularly aggressive and chemoresistant breast cancer subtype. Our findings indicate that bacopaside II exerts anti-cancer effects on TNBC cells and improves doxorubicin efficacy, mainly by increasing intracellular drug accumulation and potentially inhibiting ABC transporter-mediated drug efflux. These results position bacopaside II as a promising adjuvant in TNBC treatment, particularly for addressing resistance mechanisms that limit doxorubicin’s therapeutic effectiveness. 

Our results demonstrate that bacopaside II effectively inhibits growth in a panel of TNBC cell lines, representing different TNBC subtypes. Bacopaside II induced apoptosis in relatively sensitive cell lines, while in more resistant lines, it triggered necrosis, evidenced by membrane integrity loss. These findings suggest bacopaside II exerts multiple cytotoxic effects depending on the cellular context, an advantageous trait in treating heterogenous tumors such as TNBC. Our findings support previous reports on the anti-proliferative and pro-apoptotic effects of bacopaside II, expanding its relevance to breast cancer, particularly TNBC, which lacks specific targeted therapies [20,21,22,23]. 

A significant finding from this study is that bacopaside II increases intracellular doxorubicin accumulation, thereby enhancing doxorubicin’s cytotoxic effects across TNBC subtypes. TNBC chemoresistance is often mediated by ABC transporters, which facilitate the efflux of drugs such as doxorubicin out of cells, reducing their cytotoxic efficacy [10,11,12,13]. The increase in doxorubicin accumulation occurred independently of P-glycoprotein transcript (*ABCB1*) levels, which were low or undetectable in most TNBC cell lines except DU4475. Our docking studies suggest bacopaside II may inhibit multiple ABC transporters, including ABCB1, ABCC1, ABCC3, and ABCG2, which are implicated in TNBC chemoresistance. Moreover, the findings suggest that bacopaside II may act by interfering with substrate loading into the transmembrane domain and by inhibiting the ATPase activity of the nucleotide-binding domain [18]. The ability of bacopaside II to increase doxorubicin accumulation across various TNBC subtypes underscores its potential as a broad-spectrum modulator of chemoresistance. While these predicted interactions did not surpass those of the third-generation P-glycoprotein inhibitor elacridar, they were notably superior to cyclosporin A, a first-generation inhibitor. This suggests that bacopaside II could act as a multi-target inhibitor, potentially limiting drug efflux and sensitizing TNBC cells to chemotherapeutic agents such as doxorubicin.

In our 3D suspension culture model, bacopaside II maintained its efficacy in doxorubicin-resistant TNBC cells, which displayed stem cell-like properties and upregulated *ABCC3* and *ABCC1* transporter expression. This finding is critical, as it demonstrates that bacopaside II can retain its function in more complex tumor models that mimic the in vivo environment better than traditional 2D cultures. The sustained efficacy of bacopaside II in this model suggests it could potentially overcome acquired resistance and target cancer stem cell-like populations, which are often implicated in relapse and metastasis in TNBC.

While our findings underscore the potential of bacopaside II as an adjuvant to improve doxorubicin efficacy in TNBC, several limitations should be noted. First, our study relies on in vitro models, and in vivo studies are necessary to confirm bacopaside II’s pharmacokinetics, bioavailability, and efficacy in more complex biological systems. Notably, oral administration of bacopaside II has been shown to reduce intestinal P-glycoprotein expression and transport in animal models, indicating the need for future studies to address delivery mechanisms and tissue-specific expression profiles [19]. Additionally, while in silico docking provides insight into potential interactions between bacopaside II and ABC transporters, direct biochemical assays are required to confirm these interactions and elucidate the precise binding mechanisms.

Furthermore, as *Bacopa monnieri* is widely available in over-the-counter preparations marketed as nootropics or herbal supplements, its use alongside chemotherapy poses additional risks. These over-the-counter products often lack standardized bacopaside II concentrations, and their interaction with chemotherapeutic agents remains unknown. The potential for unintended toxicity or interference with drug metabolism necessitates caution and regulatory oversight if bacopaside II is to be repurposed as an adjuvant therapy.

To advance the clinical translation of bacopaside II, preclinical studies using animal models of TNBC are a necessary next step to assess its in vivo efficacy, toxicity, and potential for combination therapy with doxorubicin or other chemotherapeutics. Investigating its effects in patient-derived xenograft models or organoid systems may provide insights into its activity in diverse TNBC subtypes and its ability to target cancer stem cell-like populations. Furthermore, early-phase clinical trials could evaluate the safety, tolerability, and pharmacodynamics of bacopaside II in combination with existing TNBC treatments. These next steps will be crucial for determining its feasibility as a therapeutic adjuvant and its potential to improve outcomes for patients with this challenging cancer subtype.

## 5. Conclusions

In summary, our study demonstrates that bacopaside II enhances doxorubicin efficacy in TNBC by increasing intracellular drug accumulation and inhibiting ABC transporter-mediated efflux, overcoming key resistance mechanisms. These promising results underscore the need for further preclinical investigations to confirm bacopaside II’s pharmacokinetics, bioavailability, and potential to overcome resistance in vivo. Early-phase clinical trials should assess its safety, tolerability, and effectiveness in combination with doxorubicin or other chemotherapeutic agents, particularly for patients with chemoresistant or recurrent TNBC. These next steps will be crucial to translating bacopaside II into a viable adjuvant therapy to improve treatment outcomes for this challenging cancer subtype.

## Figures and Tables

**Table 1:** In silico docking analysis of bacopaside II and known P-glycoprotein (ABCB1) inhibitors, cyclosporin A and elacridar, with ABC transporter proteins.

	ABC Protein	Cyclosporin A	Elacridar	Bacopaside II
TMD	ABCB1 ABCC1 ABCC3 ABCG2	−7.324 −4.805 −2.972 −6.690	−8.177 −11.32 −10.56 −8.309	−8.361 −7.475 −6.673 −6.454
NBD	ABCB1 ABCC1 ABCC3 ABCG2	2.592 −6.779 −6.084 −5.558	−7.730 −7.514 −7.502 −7.989	−7.864 −6.864 −6.356 −7.349

Data represent estimated Gibbs free energy values (kcal/mol), reflecting the binding affinity between bacopaside II, cyclosporin A, or elacridar and the transmembrane domain (TMD) or nucleotide binding domain (NBD) of different ABC proteins.

## Data Availability

The raw data supporting the conclusions of this article will be made available by the authors on request.

## Supplementary Materials

The following supporting information can be downloaded at: https://www.mdpi.com/article/10.3390/biom15010055/s1, Figure S1: Kinetic live-cell imaging of MDA-MB-231 cell growth in response to increasing concentrations of doxorubicin; Figure S2: Spectral flow cytometry analysis of intracellular doxorubicin accumulation; Figure S3: Proportion of caspase-3/7-positive and propidium iodide (PI)-positive MDA-MB-231 cells following bacopaside II treatment; Figure S4: Western blots for MLKL and pMLKL; Figure S5: Effect of bacopaside II on doxorubicin accumulation. Figure S6: Induction of a stem cell-like phenotype in MDA-MB-231 cells cultured in a non-adherent 3D suspension.
