# Supplementary material for: Enhancement of Doxorubicin Efficacy by Bacopaside II in Triple-Negative Breast Cancer Cells
Source: Biomolecules. 2025 Jan 3;15(1):55. doi: 10.3390/biom15010055 (PMC11762400; doi:10.3390/biom15010055)
Supplement: Supplementary file 1 [file biomolecules-15-00055-s001.zip › biomolecules-3380052-supplementary.pdf]

Supplementary Materials

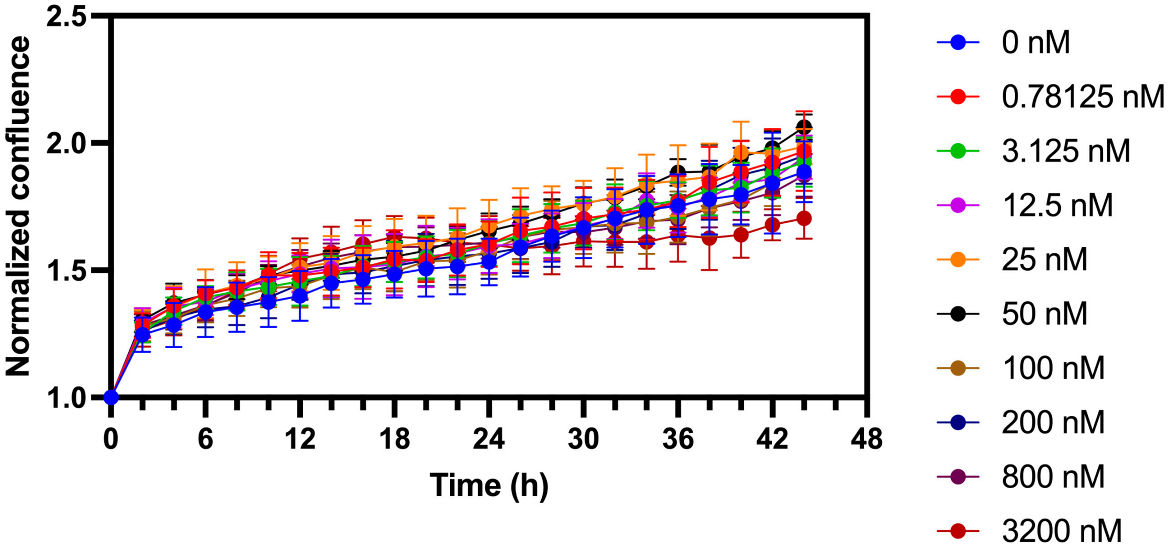

Figure S1. Kinetic live-cell imaging of MDA-MB-231 cell growth in response to increasing concentrations of doxorubicin. Confluency was measured every 2 hours over 44 hours and normalized to the confluency recorded at time 0 hours. Data represent the mean ± SD from six replicates.

(**a**)

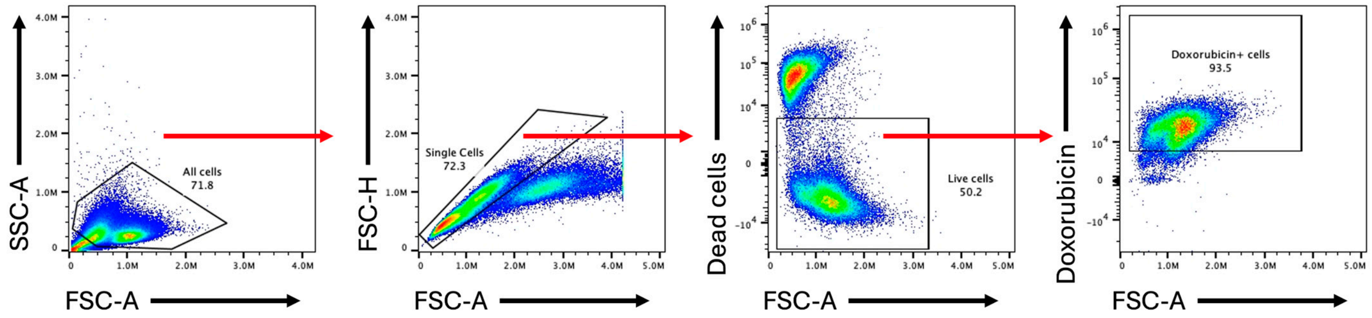

(**b**)

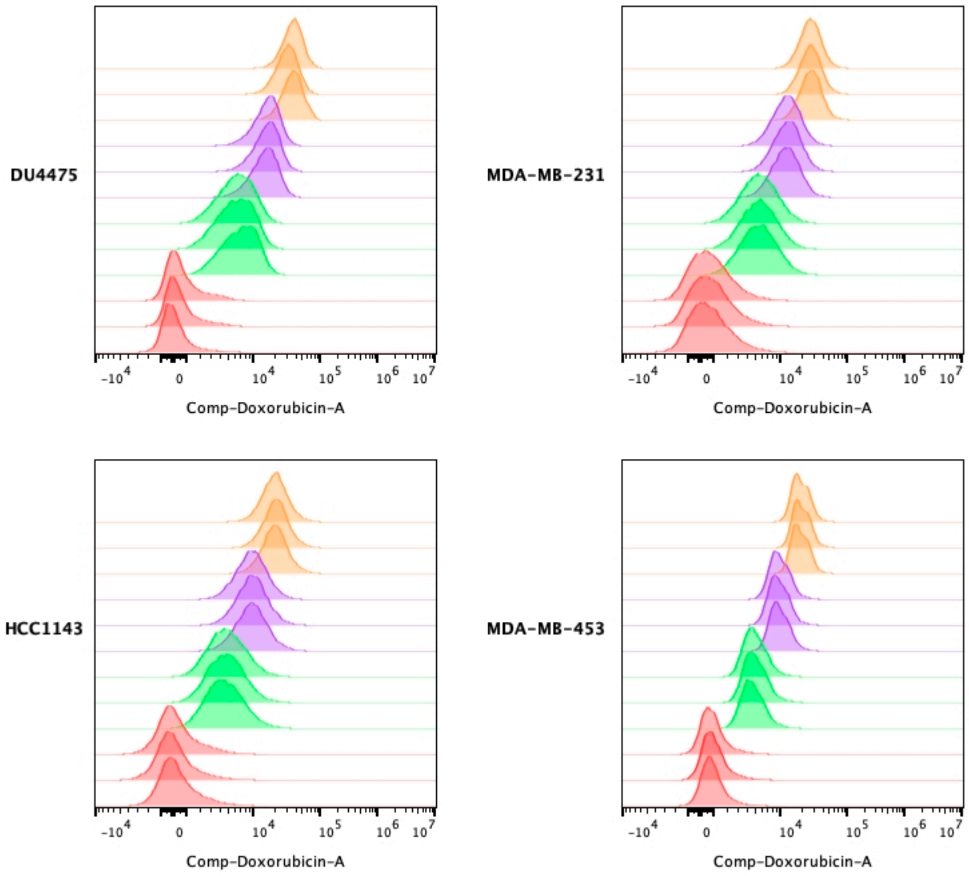

Figure S2. Spectral flow cytometry analysis of intracellular doxorubicin accumulation. (**a**) Flow cytometry gating strategy used to identify viable (live) cells and quantify intracellular doxorubicin levels. (**b**) Intracellular doxorubicin levels in viable TNBC cell lines (DU4475, MDA-MB-231, MDA-MB-453, and HCC1143) after 24 hours of treatment with increasing concentrations of doxorubicin: 0 nM (red), 25 nM (green), 50 nM (purple), and 100 nM (orange).

Figure S3

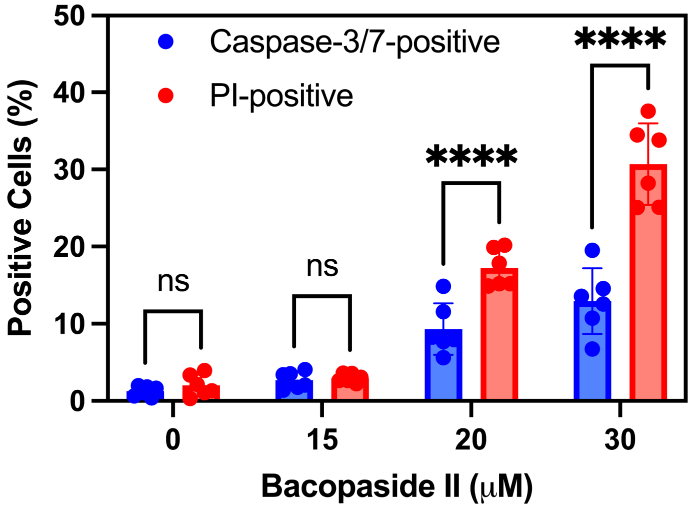

Figure S3. Proportion of caspase-3/7-positive and propidium iodide (PI)-positive MDA-MB-231 cells following bacopaside II treatment. The percentages of caspase-3/7-positive (apoptotic) and PI-positive (necrotic) MDA-MB-231 cells were measured after 6 hours of treatment with 0 μM (2% methanol vehicle control), 15 μM, 20 μM, and 30 μM bacopaside II. Data represent the mean ± SD from six replicates. Statistical significance was determined by two-way ANOVA with Sidak's multiple comparisons test; **** $p < 0.0001$.

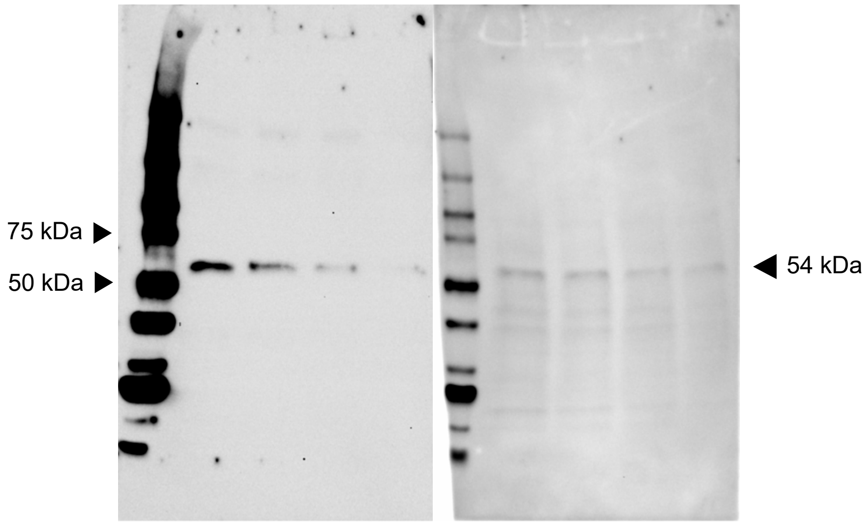

Figure S4. Western blots for MLKL and pMLKL. MDA-MB-231 cells were treated for 4 hours with 0 μM (lane 1), 15 μM (lane 2), 20 μM (lane 3), and 30 μM (lane 4) bacopaside II. A total of 50 μg of protein was loaded per lane. Membranes were probed with primary antibodies specific for MLKL (left) and phosphorylated MLKL (pMLKL, right). The expected molecular weight for both MLKL and pMLKL is 54 kDa.

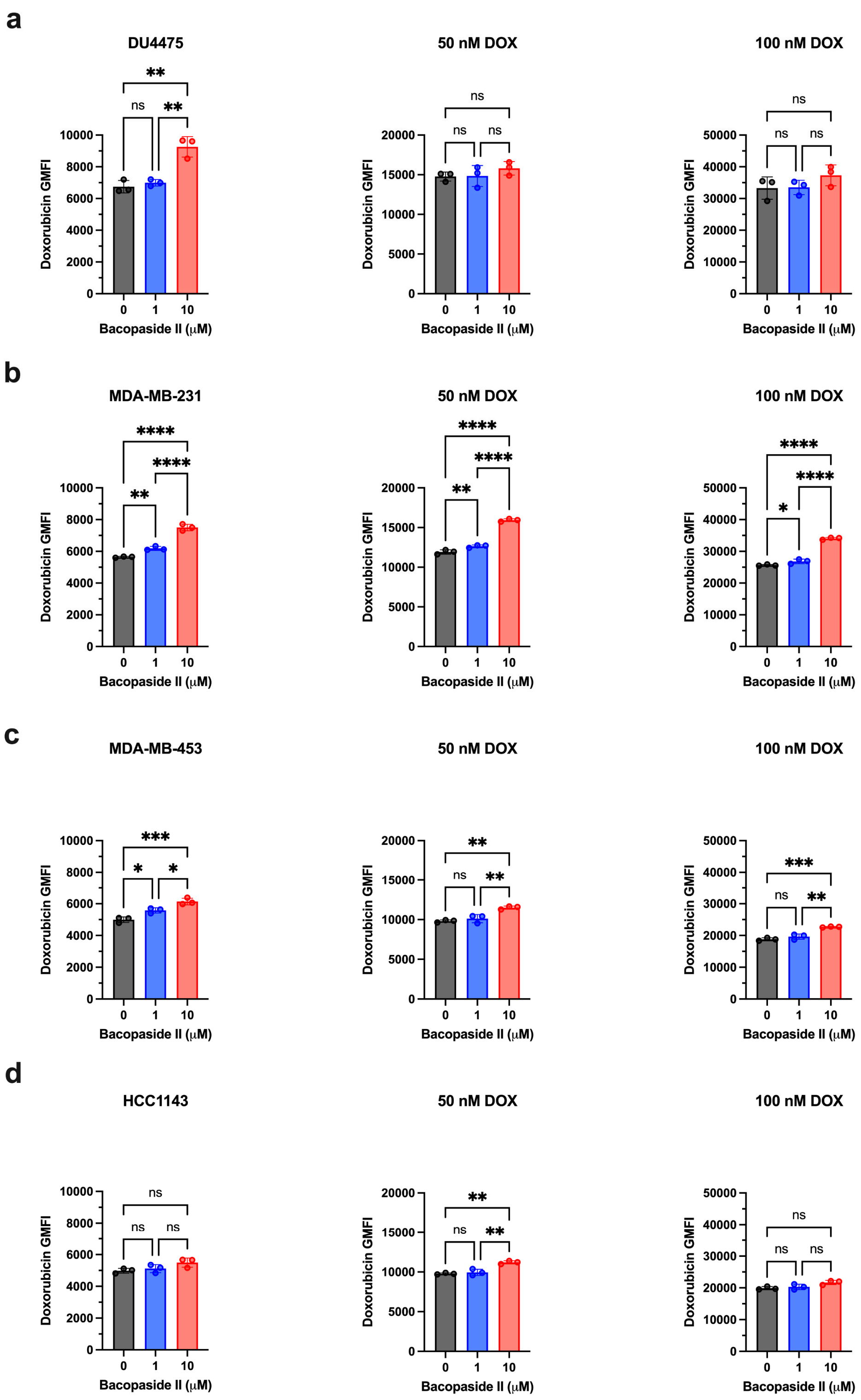

Figure S5. Effect of bacopaside II on doxorubicin accumulation. The TNBC cell lines DU4475 (**a**), MDA-MB-231 (**b**), MDA-MB-453 (**c**), and HCC1143 (**d**) were co-treated with 0 μM, 1 μM, or 10 μM bacopaside II alongside 25 nM, 50 nM, or 100 nM doxorubicin for 24 hours. Intracellular doxorubicin levels in viable cells were quantified using flow cytometry. Data represent the mean ± SD of doxorubicin geometric mean fluorescence intensity (GMFI) from three individual experiments. Statistical significance was calculated using ordinary one-way ANOVA followed by Holm-Sidak's multiple comparisons test. * $p < 0.05$, ** $p < 0.01$, *** $p < 0.001$, **** $p < 0.0001$.

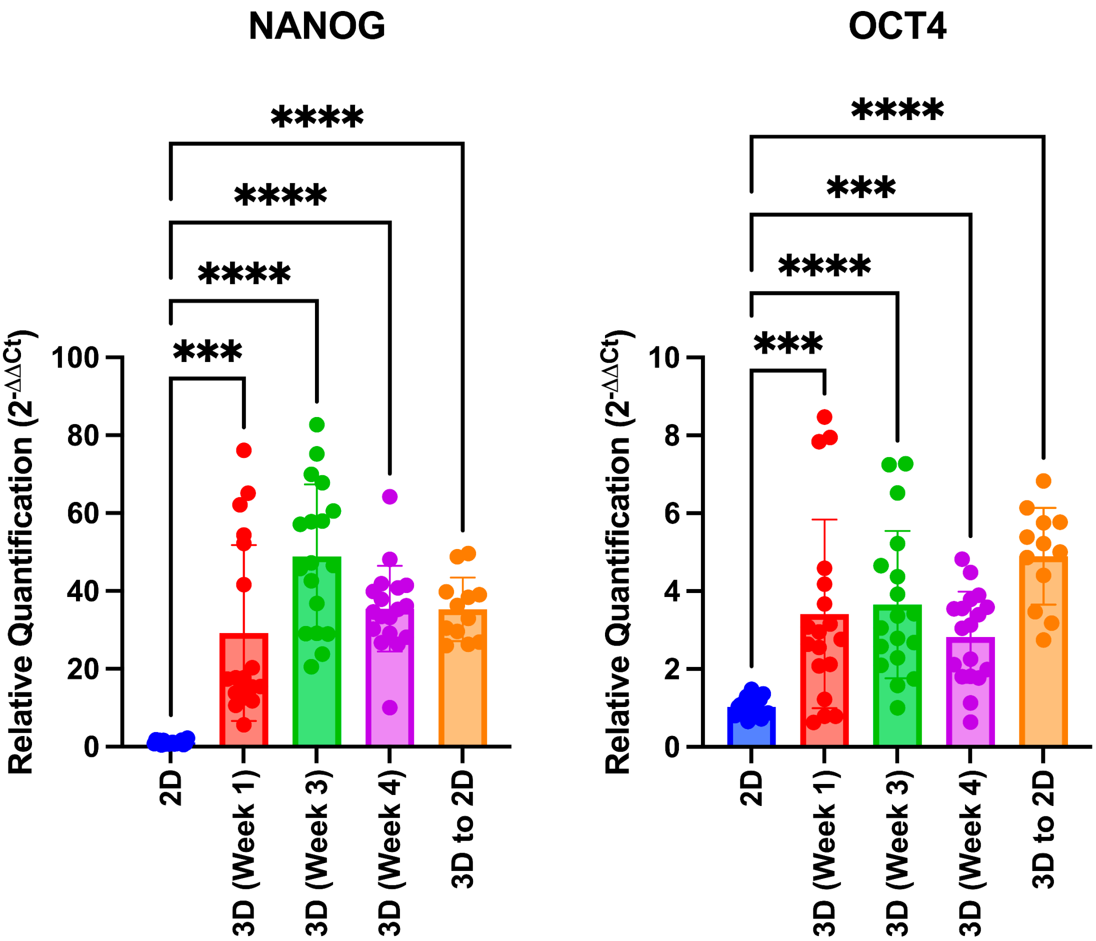

Figure S6. Induction of a stem cell-like phenotype in MDA-MB-231 cells cultured in non-adherent 3D suspension. The expression of stem cell markers *NANOG* and *OCT4* was assessed in MDA-MB-231 cells cultured under conventional adherent 2D conditions, scaffold-free 3D suspension for one to four weeks, and after reverting from 3D suspension back to adherent 2D conditions (3D to 2D). Gene expression was normalized to the *HPRT1* reference gene and calculated relative to the 2D adherent condition using the $2^{-\Delta\Delta Ct}$ method. Data are presented as mean ± SD from three technical replicates across three independent cultures. Statistical significance was determined by Kruskal-Wallis test with Dunn's multiple comparisons test; *** $p < 0.001$, **** $p < 0.0001$.
